# Measurements of Dielectric Properties of Yttrium-Stabilized Zirconia Employing Spherical Dielectric Resonator Technique

**DOI:** 10.3390/ma19183953

**Published:** 2026-09-17

**Authors:** Piotr Czekała, Adam Pacewicz, Jerzy Krupka, Krzysztof Derzakowski, Adam Abramowicz

**Affiliations:** 1 Institute of Radioelectronics and Multimedia Technology, Warsaw University of Technology, 00-665 Warsaw, Poland; adam.pacewicz@pw.edu.pl (A.P.); krzysztof.derzakowski@pw.edu.pl (K.D.); 2 Institute of Microelectronics and Optoelectronics, Warsaw University of Technology, 00-662 Warsaw, Poland; jerzy.krupka@pw.edu.pl; 3 Institute of Electronic Systems, Warsaw University of Technology, 00-665 Warsaw, Poland; adam.abramowicz@pw.edu.pl

**Keywords:** yttria-stabilized zirconia, complex permittivity, dielectric resonator method, microwave characterization, temperature dependence, spherical samples

## Abstract

Yttria-stabilized zirconia (YSZ) is a mechanically robust ceramic of interest for high-temperature microwave components and other applications requiring stable dielectric properties. However, reliable microwave permittivity data are difficult to establish due to strong dependence on composition, sample geometry, and the inherently limited sensitivity of broadband techniques for low-loss, high-permittivity dielectrics. This work presents the first application of the spherical dielectric resonator method, based on a recently formulated simplified electrodynamic model, to a non-magnetic dielectric material, extending the characterization across a broad temperature range. Spherical YSZ samples with 3.08 mol% Y_2_O_3_ content were characterized over the 5–17 GHz frequency range at room temperature and as a function of temperature from 20 °C to over 330 °C using dielectric resonator measurements in five different cylindrical cavities with a high electric energy filling factor in the sample ($p_{e}\approx 0.96$). Complex permittivity was extracted with a simplified equivalent spherical-enclosure model and cross-checked for a representative case using a radial mode-matching approach. At room temperature, the extracted real part of permittivity was consistent across the investigated band, while the dielectric loss tangent was on the order of $10^{-3}$ and showed a slight increase with frequency. Temperature measurements indicated that both real part of permittivity and dielectric loss tangent increase monotonically with temperature. The obtained results provide a consistent microwave dataset for polycrystalline YSZ.

## 1. Introduction

Yttria-stabilized zirconia (YSZ) (ZrO_2_)_x_(Y_2_O_3_)_1−x_ is one of the mechanically strongest commercially available ceramic materials. It is used at harsh environment conditions in ball grinding mills, in plasma coatings, as a refractory material in jet engines, and in microwave electronics. Its unique physical properties include extremely high strength and toughness, very high impact resistance, high erosion and corrosion resistance, high permittivity, and low dielectric losses [1].

At room temperature, its ionic conductivity is very small, but it increases to about $1\times 10^{-2}$ S/m at 520 °C [2]. It is well-suited for microwave devices operating at extremely high-temperatures such as patch antennas [3]. Patch antennas made of YSZ would operate at the fire services communication and can be installed in flying, through the atmosphere, objects such as space ships or ballistic missiles. For microwave applications, important material properties of YSZ are the real part of permittivity ($\varepsilon_{r}^{\prime }$), the dielectric loss tangent (tan δ), the thermal coefficient of resonance frequency and the thermal coefficient of permittivity. Another application where $\varepsilon_{r}^{\prime }$ and tan δ at microwave frequencies would be of interest are materials for the next generation semiconductor devices where high permittivity dielectric layers are used to replace the traditional low permittivity SiO_2_ gate dielectric [4,5].

Although numerous papers have been devoted to measurements of the dielectric properties of YSZ, e.g., [6,7,8,9,10,11,12], it is difficult to establish accurate values of these parameters based on the literature data. It is well known that the permittivity of YSZ strongly depends on the yttria content [1] and, as for all ceramics, on the density of the sample. Real permittivity values of YSZ measured up to microwave frequencies reported in the literature vary in the range of 4–40 [8,12], while the dielectric loss tangent values vary by one order of magnitude. This is mostly because of the material structure (crystallographic phases of YSZ depend on doping and the temperature of operation), but partly due to inadequate methods employed in the loss tangent measurements. For instance, transmission/reflection methods have limited loss tangent resolution on the order of 10^−2^ and are very sensitive to the presence of air gaps [13]. For low-loss materials, this can lead to large uncertainties or even non-physical outcomes, such as negative loss tangent values. Therefore, in order to accurately characterize YSZ losses, a method with significantly higher loss resolution is required. An example of such a method is the dielectric resonator technique, which is considered to be one of the most accurate methods for the microwave characterization of low-loss dielectrics [13,14]. In such methods, cylindrical or ring-shaped dielectric samples are placed inside a metallic resonant cavity, and their complex permittivity is extracted based on the measured resonant frequency and quality factor [7,13]. However, commercially available YSZ samples are typically spherical, which makes it impossible to use the dielectric resonator method in its original form. Notably, in [15], a new formulation of this method, aimed at the characterization of spherical ferrite samples, was presented. In this work, the same method is used to characterize spherical YSZ samples in the 5–17 GHz range. Moreover, since the dielectric resonator method can also be successfully used in thermal measurements, as presented, e.g., in [16], the YSZ samples were additionally characterized as a function of temperature, providing a robust dataset as a function of both frequency and temperature. The latter is particularly important given the high-temperature applications of YSZ.

To date, the dielectric resonator technique has been applied mainly to cylindrical or ring-shaped samples [7,13,14]. Characterization of samples with significantly different geometries, such as spheres, has remained impractical because the standard electromagnetic formulations do not account for the spherical boundary. A simplified electrodynamic model for spherical dielectric resonators was recently introduced in [15] and validated for ferrite samples. However, its applicability to non-magnetic dielectrics ($\mu_{r}$ = 1) and its performance over extended temperature ranges have not been investigated. The present work addresses this gap by applying the simplified model to spherical YSZ samples across a broad frequency range (5–17 GHz) and a wide temperature range (20–336 °C). We demonstrate that the resulting measurement uncertainties are comparable to those achieved with conventional cylindrical-sample configurations, establishing the method as a reliable tool for the microwave characterization of spherical dielectric specimens.

In this work, it is shown that a dielectric resonator-based approach used previously for cylindrical-shaped samples can be successfully extended to spherical YSZ samples, enabling repeatable extraction of complex permittivity over the 5–17 GHz frequency range and across temperature, starting from 20 °C up to over 330 °C. Importantly, such temperature-dependent microwave characterization provides much more than a single “room-temperature” result: the frequency and temperature dependence of $\varepsilon_{r}^{\prime }$ directly govern resonance-frequency drift, impedance matching, and dimensional scaling of microwave components, while tan δ determines the Q-factor, insertion loss and efficiency, especially under thermal loading. Therefore, the proposed dataset enables reliable design and modeling of YSZ-based microwave structures operating in harsh and high-temperature environments, and supports predictive electro-thermal simulations where accurate $\varepsilon_{r}^{\prime }$(f,T) and tan δ(f,T) are required inputs. The resulting dataset reduces the spread of literature values and provides a reliable reference for high-temperature microwave components and high-permittivity (“high-k”) dielectric applications.

## 2. Materials and Methods

### 2.1. Electromagnetic Model

**Figure 1:**
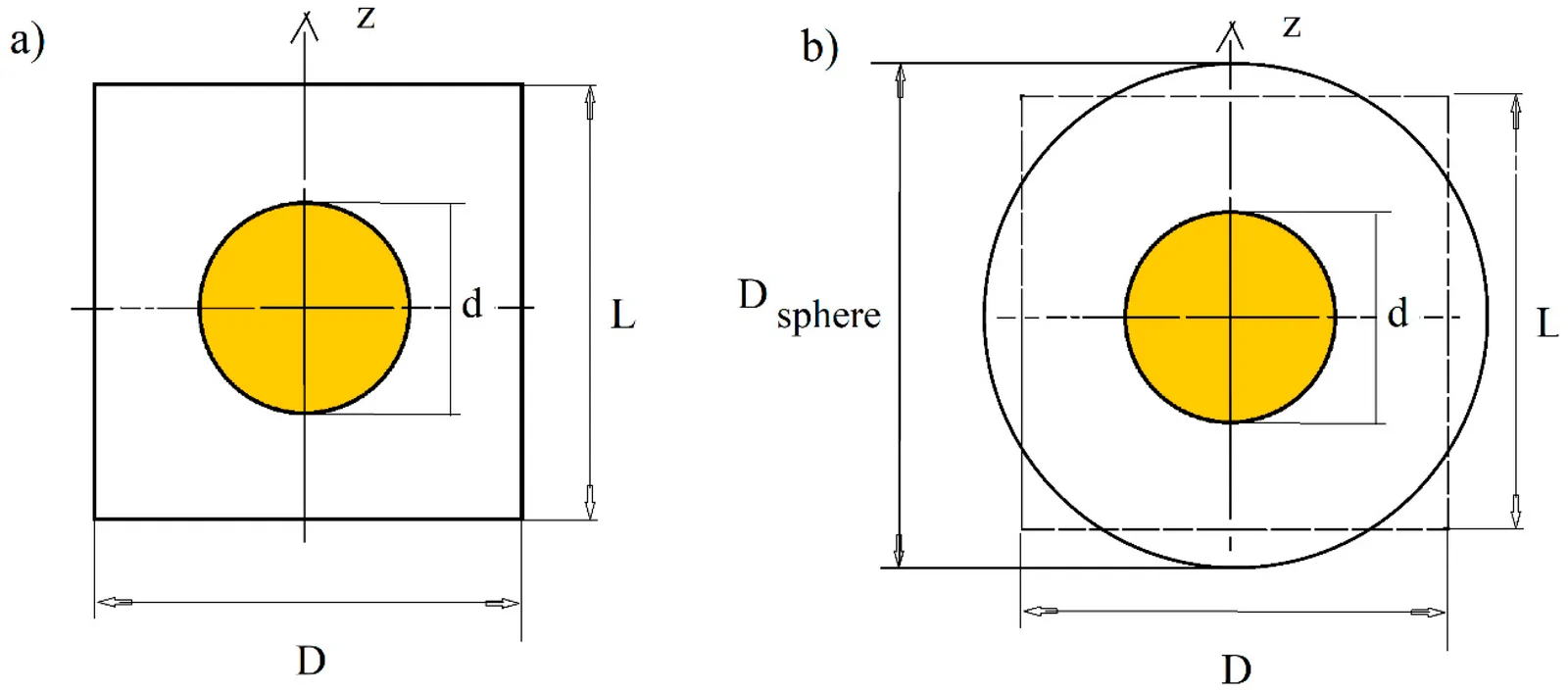
(**a**) Cylindrical cavity containing a spherical sample (orange) placed at its center, with the cavity walls indicated by a solid black line; (**b**) geometry of the simplified spherical model.

Measurements of spherical samples were performed using five cylindrical resonant cavities with different diameters and heights (covering different frequency bands). As shown in [15], a simplified electrodynamic model of the cylindrical resonant structure can be used for numerical analysis with an accuracy significantly better than the experimental uncertainties. The concept of this simplification is presented in Figure 1. In brief, the original cylindrical cavity containing a spherical sample at its center (Figure 1a) is replaced by an equivalent spherical cavity (Figure 1b). This substitution reduces the electromagnetic problem from 2D to 1D, thereby considerably simplifying the numerical calculations.

The effective diameter of the spherical cavity, $D_{sphere}$, is chosen such that the resonance frequency of the TE_101_ mode of the empty spherical cavity equals the resonance frequency of the TE_011_ mode of the empty cylindrical cavity. The resulting effective diameter is given by:

(1) $$D_{sphere}=\frac{2u_{0}}{\sqrt{{\left(\frac{2v_{0}}{D}\right)}^{2}+{\left(\frac{\pi }{L}\right)}^{2}}}$$

where D is the cylindrical cavity diameter and L is the cavity height. The constants $u_{0}\approx 4.49341$ and $v_{0}\approx 3.83171$ are the first roots of the Bessel functions $J_{3/2}$ and $J_{1}$, respectively, i.e., $J_{3/2}\left(u_{0}\right)=0$ and $J_{1}\left(v_{0}\right)=0$.

To position the sample at the cavity center, a mechanical support is required. If the support is sufficiently small and made of a material with much lower $\varepsilon_{r}^{\prime }$ than that of the sample, its influence on the resonance frequency is negligible and, consequently, it can be omitted in the simplified model. For the YSZ samples analyzed in this work, numerical analysis using the radial mode-matching method [17] showed that neglecting the support changes the resonance frequency by less than 0.16%, which is negligible compared to other sources of measurement uncertainty discussed later.

In the process of extracting the complex permittivity of the sample under test, the following parameters must be numerically determined: the resonance frequency, the electric energy filling factor in the sample, and the geometric factor of the resonance cavity. The general procedure for determining the complex permittivity of low-loss materials is as follows. First, the real part of permittivity is determined from the following relationship:

(2) $$F(f_{meas},\;d,\;\varepsilon_{r}^{\prime },D_{sphere})=0$$

where F denotes the relationship between the resonance frequency of a specific mode $f_{meas}$ and other parameters of a model such as $\varepsilon_{r}^{\prime }$ and dimensions d and $D_{sphere}$. For the resonance structure in Figure 1b this relationship is given as a transcendental equation which can be found in [18].

Once the real part of permittivity is determined, the dielectric loss tangent is evaluated from the formula:

(3) $$tan\;\delta =\frac{1}{p_{e}Q_{d}}$$

where $p_{e}$ denotes the electric energy filling factor in the sample and $Q_{d}$ is the Q-factor associated with dielectric losses of the sample:

(4) $$Q_{d}={\left(\frac{1}{Q_{u}}-\frac{1}{Q_{c}}\right)}^{-1}$$

where $Q_{u}$ is the measured unloaded Q-factor of the resonance structure, $Q_{c}$ is the Q-factor depending on conductor losses in the metal enclosure:

(5) $$Q_{c}=\frac{G}{R_{s}}.$$

G in Equation (5) denotes the computed geometric factor and $R_{s}$ is the surface resistance of metal walls at the given frequency $f_{meas}$. The surface resistance can be evaluated, based on the measurements of the empty cavity Q-factor. Radiation losses are neglected in Equation (4) owing to the closed geometry of the resonant structure. Moreover, dielectric losses associated with the sample supports—made of expanded polystyrene, single-crystal quartz, or fused silica—are likewise negligible due to their very low electric energy filling factors and very low intrinsic loss tangents.

The theoretical model described above has already been validated employing an electromagnetic simulator (COMSOL 6.2) and experiments with spherical magnetic garnet samples [15]. Commercially available full-wave electromagnetic simulators such as HFSS, COMSOL, and QuickWave allow for computations of the resonance frequencies of complicated structures for given material properties of the structure. However, their generality comes at the cost of substantially longer computation time compared with semi-analytical solvers dedicated to a specific class of resonant structures, without a corresponding improvement in accuracy for this problem [17]. Consequently, employing full-wave solvers to address the inverse problem (i.e., extracting complex permittivity from measured resonance data) results in impractically long computation times for materials characterization.

**Figure 2:**
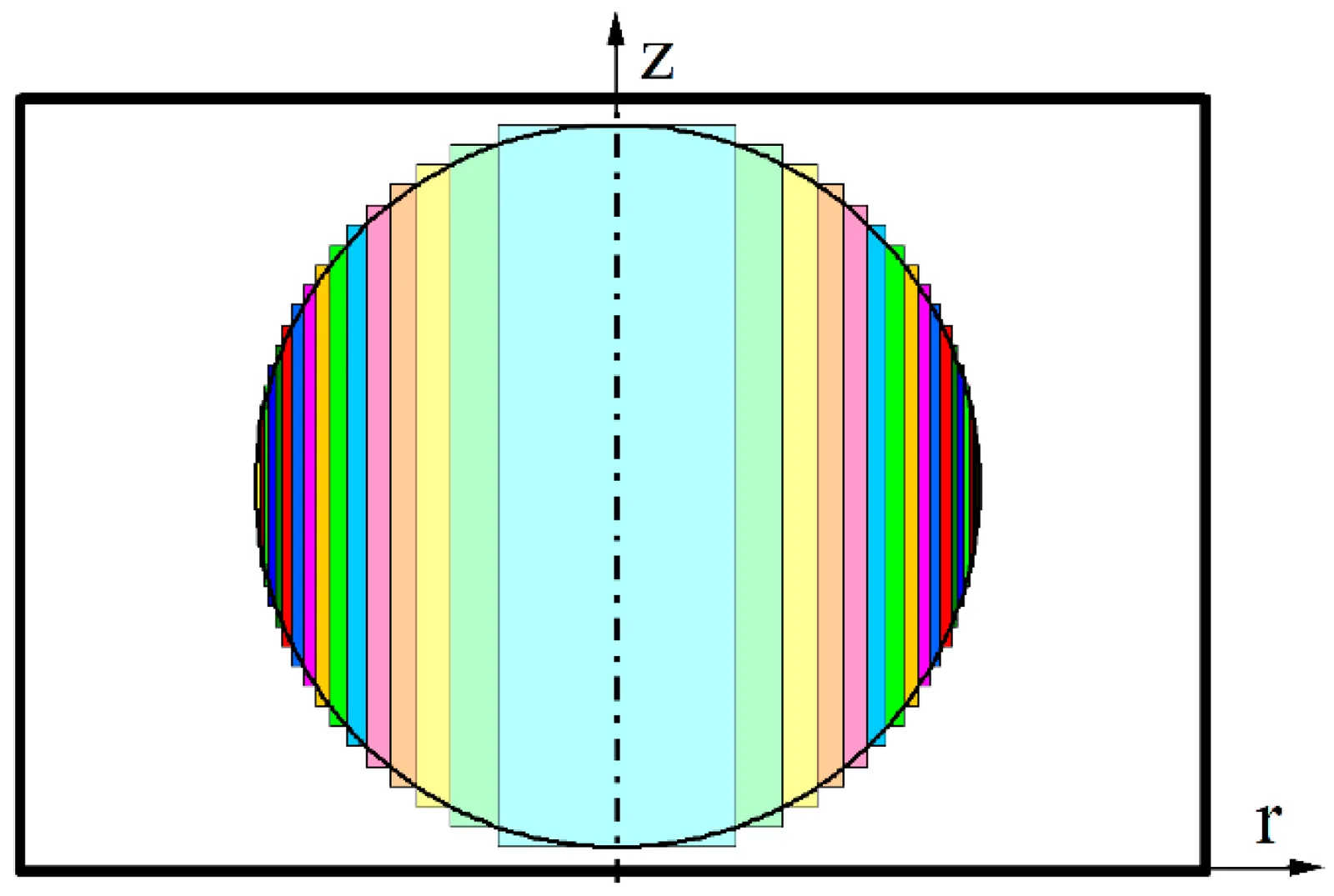
Geometry of the resonance structure which was used for the radial mode-matching analysis for the stack of cylinders circumscribed by a sphere. The sphere is divided in the radial mode-matching method into 18 regions.

The resonant structure shown in Figure 1a was additionally analyzed using the radial mode-matching (RMM) technique, described in detail in [17]. For the RMM analysis, the spherical sample was approximated by a stack of coaxial cylinders. The corresponding geometry is illustrated in Figure 2 for the case of a cylinder stack circumscribed by a sphere. For a given material permittivity, the resonance frequency of the quasi-TE_011_ mode obtained for the circumscribed-cylinder approximation is expected to be lower than that of the true spherical sample. This is because the volume of the cylinder stack used to represent the sample is larger than the volume of the sphere, which increases the electric energy filling factor and shifts the resonance frequency downward. Therefore, the RMM result based on the circumscribed-cylinder geometry can be treated as a lower bound for the resonance frequency of the structure shown in Figure 1a. To obtain an upper bound, an alternative approximation based on a stack of cylinders inscribed in the sphere is used in the computations.

Validation of the simplified electromagnetic model (Figure 1b) against the RMM approach was performed by comparing resonance frequencies obtained with RMM for the two spherical approximations (cylinder stacks circumscribed by a sphere and inscribed in a sphere). In this comparison, the $\varepsilon_{r}^{\prime }$ value used in the RMM calculations was set to the value extracted with the simplified model from the measured resonance frequency. Specifically, we analyzed a cylindrical cavity with diameter D = 24 mm and height L = 12 mm, loaded with a YSZ sample (3.08 mol% Y_2_O_3_ content) of diameter d = 4.95 mm. The measured resonance frequency was $f_{meas}=$ 10.264752 GHz and the corresponding extracted permittivity was $\varepsilon_{r}^{\prime }$ = 33.510. When this $\varepsilon_{r}^{\prime }$ value was used as an input to the RMM solver, the following resonance frequencies were obtained: $f_{r,min}=$ 10.17064221 GHz for the circumscribed-cylinder approximation and $f_{r,max}=$ 10.41984876 GHz for the inscribed-cylinder approximation. These values create the upper and the lower bound of the measured frequency so our model, where we substituted cylindrical cavity with the equivalent spherical cavity (Figure 1b), has been validated.

### 2.2. Samples and Measurement Setup

The experimental campaign comprised two main parts. First, several spherical YSZ samples were characterized at room temperature (T=24±1 °C) using five different cylindrical resonant cavities, which enabled determination of the complex permittivity as a function of frequency in the 5–17 GHz range. Second, two selected samples were characterized as a function of temperature to quantify the temperature dependence of the dielectric parameters of YSZ. Four cavities were fabricated from copper and subsequently silver-plated, while the cavity operating at 5.423 GHz was made of molybdenum. The cavity dimensions were selected to ensure a high electric-field energy filling factor in the sample, thereby increasing the sensitivity of the resonance frequency to the real part of permittivity and the sensitivity of the unloaded quality factor to the dielectric loss tangent.

Spherical YSZ samples were obtained from a commercial supplier of ceramics (Xiamen Wintrustek Advanced Materials Co., Ltd., Xiamen, China, Product No.: WTKZRO-2507004). The samples are classified as yttria-stabilized tetragonal zirconia polycrystal (Y-TZP) grade, with specified density 6.0 g/cm^3^ and grain size < 0.5 µm. All the samples were specified to contain approximately 3.08 mol% Y_2_O_3_. The nominal diameters were approximately 3 mm, 5 mm, and 9.5 mm. The actual diameters of the samples used in this study were measured independently using a micrometer screw gauge. The measured deviations from nominal diameter and from perfect sphericity did not exceed 1% for any of the samples listed in Table 1. Direct experimental verification of the chemical composition (e.g., XRF or EDS) and phase identification (XRD) were not performed in this study. However, the phase composition can be reliably inferred from the yttria content: according to the Y_2_O_3_–ZrO_2_ phase diagram presented by Graeve [1], the tetragonal phase is stable at room temperature for yttria concentrations in the approximate range of 2–8 mol% in well-densified materials, placing the 3.08 mol% composition clearly within the tetragonal phase field. This assignment is consistent with the Y-TZP grade designation and with the high permittivity and low loss tangent measured in this work.

During room-temperature measurements, the samples were positioned at the cavity center using Styrofoam supports ($\varepsilon_{r}^{\prime }\approx 1.02$). Owing to the low permittivity and small size of the support, its influence on the measured resonance frequency was negligible and was therefore omitted in the simplified model.

The resonance frequencies and unloaded Q-factors were measured using a vector network analyzer (VNA, Keysight PNA-X N5245A). The resonators were excited via coaxial couplings, and the transmission response was recorded in the $S_{21}$ configuration. The coupling loops were set in such a way to provide weak and symmetrical coupling, thanks to which the unloaded quality factor can be calculated based on the measured loaded quality factor, $Q_{l}$, using the standard decoupling formula [19]:

(6) $$Q_{u}=\frac{Q_{l}}{1-|S_{21}(f_{r})|},$$

where $\left|S_{21}(f_{r})\right|$ denotes the magnitude of the transmission coefficient at the resonant frequency, expressed in linear units. To reduce the measurement uncertainty, the resonant frequencies and the unloaded quality factors were extracted from the recorded S_21_ curves using a complex resonance-curve fitting procedure [19]. Since the unloaded quality factor defined by Equation (6) inherently accounts for coupling losses [19], these losses need not be explicitly included in Equation (4).

### 2.3. Thermal Measurements

**Figure 3:**
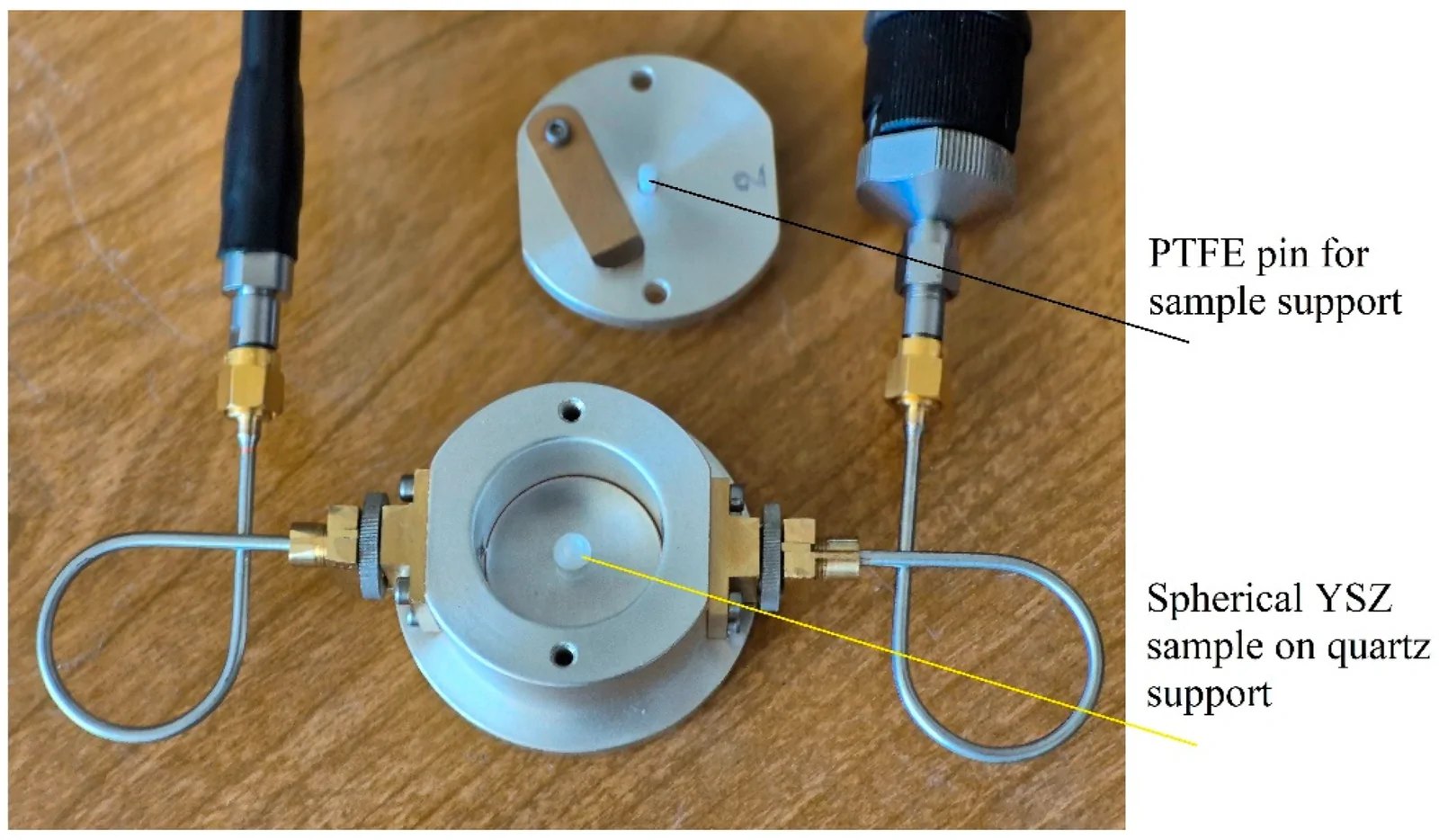
Photograph of D = 24 mm, L = 12 mm copper–silver plated cavity containing d = 4.95 mm YSZ sample, situated on a small quartz support having a height of 3.5 mm.

Two sets of temperature-dependent measurements were performed. The first set covered the range 20–90 °C (293.15–363.15 K) in 5 °C steps using the copper, silver-plated cavity with D = 24 mm, L = 12 mm (Figure 3) loaded with a YSZ sphere of diameter d = 4.95 mm. The resonator was placed in an environmental chamber at a relative humidity of 30%. Because the chamber introduced mechanical vibrations, the sample was supported using a small single-crystal quartz support, and a spring-loaded PTFE pin was applied from the top to maintain a fixed position of the spherical sample during the measurements (Figure 3). The temperature was monitored using the environmental chamber sensor. At each temperature step, the system was allowed to stabilize for approximately 90 min prior to data acquisition.

**Figure 4:**
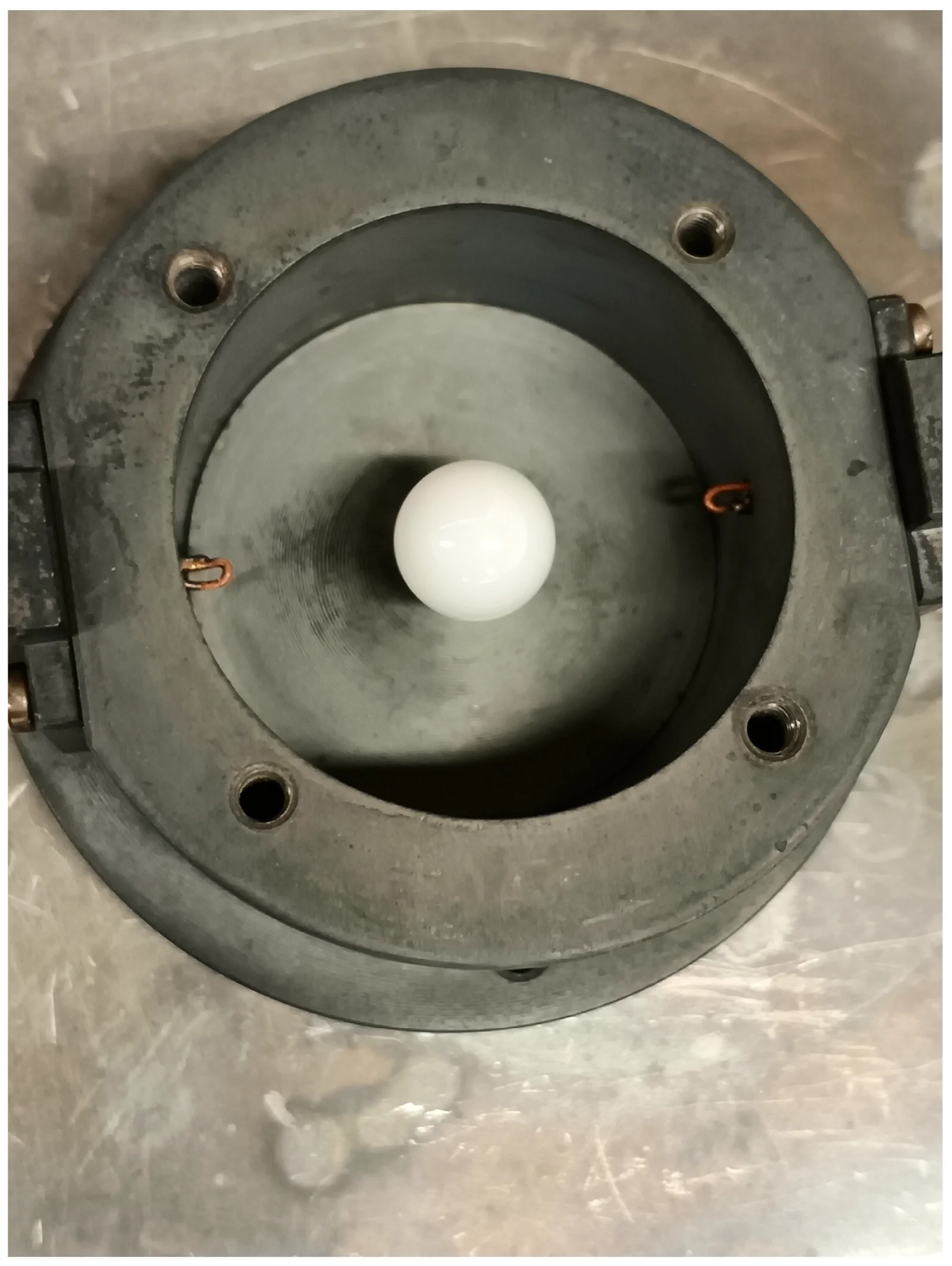
Photograph of D = 34 mm, L = 17 mm molybdenum cavity containing d = 9.52 mm YSZ sample, situated on a small fused silica support having height of 5.0 mm.

The second set of thermal measurements covered the range 23–336 °C (296.15–609.15 K) using the molybdenum cavity with D=34 mm and L=17 mm (Figure 4) loaded with a YSZ sphere of diameter d=9.52 mm. The cavity was placed in a tube furnace capable of heating up to 1100 °C. Measurements were limited to approximately 336 °C because at higher temperatures, the resonance Q-factor became too low to reliably determine the resonance frequency, and Q as the resonance curve became very broad and non-Lorentzian. The temperature was monitored using the furnace built-in thermocouple, and the uncertainty of the set temperature was approximately ±1 °C.

Thermal expansion of the sample was taken into account in the extraction of $\varepsilon_{r}^{\prime }$ and tan δ. The temperature-dependent linear thermal expansion coefficient α(T) of YSZ was taken from the measurements reported by Hayashi et al. for various yttria contents [20]. The data were interpolated to 3.08 mol% yttria contents and approximated using a polynomial fit α(T) over the considered temperature range. The sample diameter at a given temperature $T_{k}$, $d(T_{k})$, was then calculated from the room-temperature diameter $d(T_{0})$ as:

(7) $$d\left(T_{k}\right)=d\left(T_{0}\right)\left[1+\int_{T_{0}}^{T_{k}}\alpha \left(T\right)\;dT\right]$$

where $T_{0}$ is the room temperature at which the diameter of the sample was measured.

Using the measured resonance frequency f(T) and the extracted real part of permittivity $\varepsilon_{r}^{\prime }(T)$, the thermal coefficient of resonance frequency (TCF) and the thermal coefficient of permittivity ($\alpha_{\varepsilon }$) were calculated as:

(8) $$TCF=\;\frac{1}{f}\frac{\partial f}{\partial T}$$

(9) $$\alpha_{\varepsilon }=\frac{1}{\varepsilon_{r}^{\prime }}\frac{\partial \varepsilon_{r}^{\prime }}{\partial T}$$

In practice, the derivatives in Equations (8) and (9) were determined from the experimental f(T) and $\varepsilon_{r}^{\prime }(T)$ data.

## 3. Results

### 3.1. Room-Temperature Results

The extracted room-temperature values of the $\varepsilon_{r}^{\prime }$ and tan δ of YSZ are summarized in Table 1. The measurement frequency range of approximately 5–17 GHz results directly from the available sample dimensions and the inverse scaling of the dielectric resonator frequency with sample size; extending the characterization to lower frequencies would require substantially larger samples than those commercially available. The results were obtained using the simplified electromagnetic model, in which the spherical sample is analyzed in an equivalent spherical enclosure of diameter $D_{sphere}$ (Figure 1b). For the case marked with “*”, the permittivity extraction was additionally performed using the RMM technique.

In contrast to general-purpose full-wave electromagnetic solvers, which typically address the inverse problem by iteratively simulating resonance frequencies for multiple trial values of $\varepsilon_{r}^{\prime }$, the RMM approach enables direct determination of $\varepsilon_{r}^{\prime }$ from the measured resonance frequency and the sample geometry, which makes that method more suitable for the material characterization purposes.

Mode-matching results correspond to the average of the two spherical approximations, i.e., stacks of cylinders circumscribed by a sphere and inscribed in a sphere (Figure 2). Depending on the adopted cylindrical approximation, the extracted $\varepsilon_{r}^{\prime }$ varies noticeably, from $\varepsilon_{r,min}^{\prime }=32.775$ (circumscribed stack) to $\varepsilon_{r,max}^{\prime }=34.676$ (inscribed stack). The tan δ value obtained with the mode-matching technique is approximately 4.8% higher than that obtained with the simplified model, which is attributed to the fact that conductor-wall losses are not separated in the mode-matching approach and are therefore partially assigned to the dielectric loss of the sample. In contrast, the $\varepsilon_{r}^{\prime }$ values obtained with both methods differ by less than 0.7%.

The extracted room-temperature $\varepsilon_{r}^{\prime }$ values are mutually consistent, with an average $\varepsilon_{r,\;avg}^{\prime }$ = 33.474 and standard deviation σ($\varepsilon_{r}^{\prime }$) = 0.137, which is well within the estimated 2% measurement uncertainty. Within this uncertainty, no systematic frequency dependence of $\varepsilon_{r}^{\prime }$ is observed over the investigated 5–17 GHz range. It should be noted that, because the simplified electromagnetic model employed here has been validated only for the fundamental TE_01δ_ mode [15], different samples were measured at different frequency points. The small scatter of the results (σ/$\varepsilon_{r,\;avg}^{\prime }$ ≈ 0.4%) suggests, however, that sample-to-sample variation does not significantly affect the extracted permittivity values. The dielectric loss tangent shows a slight increase with frequency. The relative uncertainty of $\varepsilon_{r}^{\prime }$ is estimated to be approximately 2%, dominated by the uncertainty in the measured sample diameter (including deviations from an ideal sphere), which did not exceed 1% for the samples listed in Table 1. The uncertainty contribution associated with the electromagnetic model is considered negligible in comparison. As demonstrated in [15], the simplified model reproduces resonance frequencies and electric energy filling factors to within 0.2% and 0.5%, respectively, relative to full-wave simulations across a wide range of sample-to-cavity configurations, which translates to an uncertainty in $\varepsilon_{r}^{\prime }$ of approximately ±0.4%. The additional cross-validation against the RMM technique for one configuration (Table 1, d = 9.52 mm) further confirms the model accuracy: the average RMM result ($\varepsilon_{r}^{\prime }$ = 33.726) differs from the simplified-model value ($\varepsilon_{r}^{\prime }$ = 33.510) by only 0.6%, well within the 2% overall measurement uncertainty, while the RMM approach itself exhibits a ~6% spread between its two cylindrical approximations of the sphere. For tan δ, the dominant uncertainty source is the uncertainty of the Q-factor determination, which typically is at the level of ~1% (standard deviation) [21]. That uncertainty usually corresponds to a few percent uncertainty of the low-loss materials tan δ [21].

### 3.2. Temperature Measurement Results

**Figure 5:**
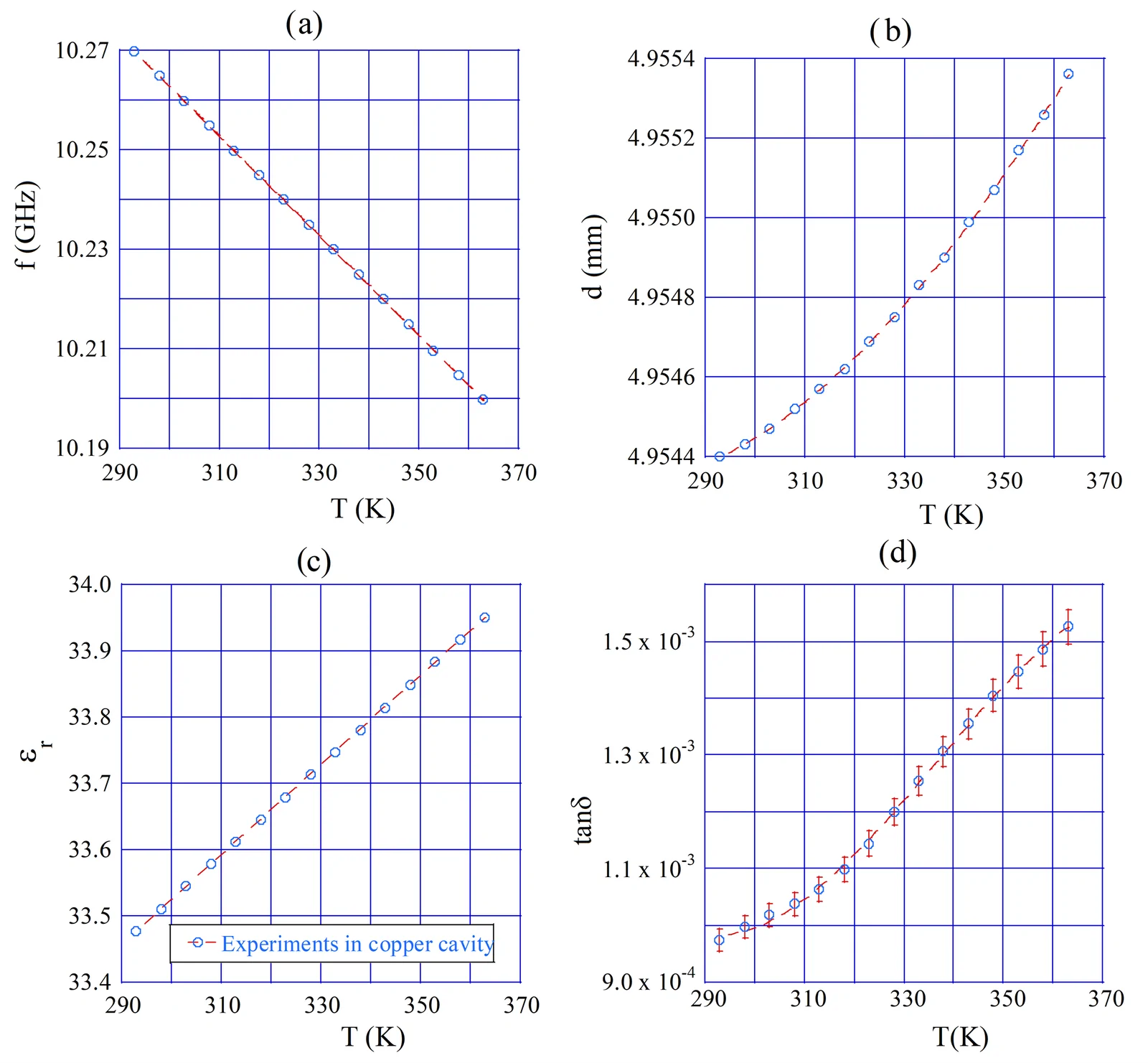
Results of measurements in copper–silver-plated cavity (D = 24 mm, L = 12 mm), presented in Figure 3: (**a**) resonance frequency versus temperature; (**b**) diameter of the sample as a function of temperature; (**c**) real part of permittivity versus temperature; (**d**) the dielectric loss tangent as a function of temperature.

Temperature-dependent measurements were carried out in two temperature intervals (20–90 °C and 23–336 °C), as described in Section 2. The results obtained in the 20–90 °C range are presented in Figure 5. Both the real part of permittivity (Figure 5c) and the dielectric loss tangent (Figure 5d) of the investigated YSZ sample ($d(T_{0})\approx 4.95$ mm) increase monotonically with temperature. The increase in $\varepsilon_{r}^{\prime }$ is nearly linear and the corresponding thermal coefficient of permittivity, calculated based on results presented in Figure 5c according to Equation (9), is $\alpha_{\varepsilon }=(2.016\pm 0.03)\times 10^{-4}\;K^{-1}$. This increase in $\varepsilon_{r}^{\prime }$ leads to a nearly linear decrease in the resonance frequency with temperature (Figure 5a), with $TCF=-(9.787\pm 0.2)\times 10^{-5}\;K^{-1}$ (Equation (8)).

**Figure 6:**
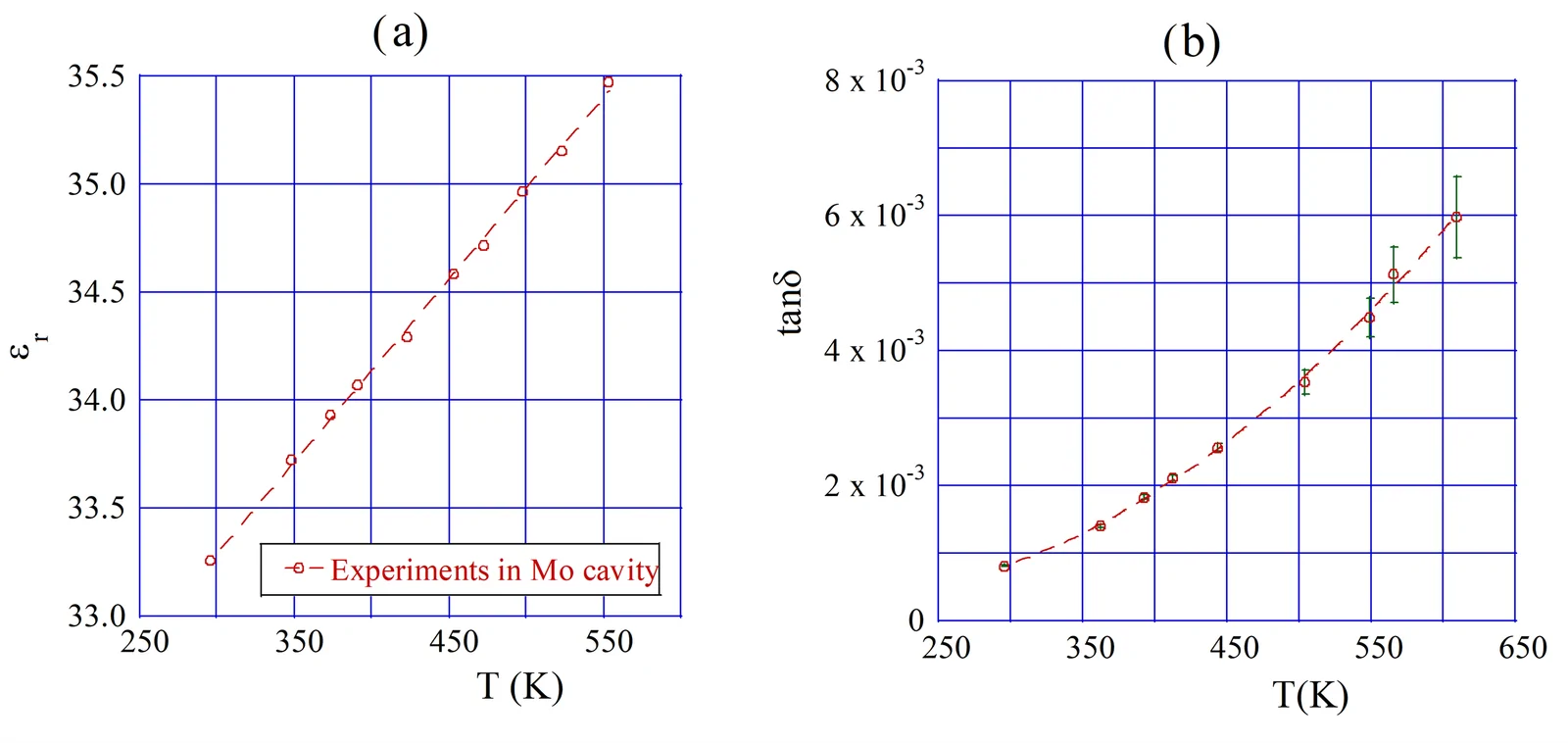
Results of measurements in molybdenum cavity (*D* = 34 mm, *L* = 17 mm) loaded with YSZ sample of diameter $d(T_{0})\approx 9.52$ mm: (**a**) real part of permittivity versus temperature; (**b**) the dielectric loss tangent as a function of temperature. The resonance frequency of the cavity at room temperature was equal to $f\left(T_{0}\right)\approx 5.423$ GHz.

The results obtained in the 23–336 °C range are presented in Figure 6. Consistent with the lower-temperature dataset (Figure 5) obtained for the $d(T_{0})\approx 4.95$ mm sample, the real part of permittivity of the $d(T_{0})\approx 9.52$ mm sample increases approximately linearly with temperature, and this trend remains valid over the entire investigated range up to 336 °C (Figure 6a). The thermal coefficient of permittivity determined from results presented in Figure 6a (Equation (9)) is $\alpha_{\varepsilon }=(2.048\pm 0.03)\times 10^{-4}\;K^{-1}$. The corresponding thermal coefficient of resonance frequency for the molybdenum cavity, calculated from f(T) using Equation (8), is $TCF=-(1.039\pm 0.02)\times 10^{-4}\;K^{-1}$. The dielectric loss tangent (Figure 6b) increases monotonically with temperature and is in good agreement with the trend observed in the 20–90 °C measurements (Figure 5d).

### 3.3. Uncertainty Analysis

The main sources of uncertainty in the determination of the dielectric parameters of spherical samples are quantified below.

#### 3.3.1. Real Part of Permittivity

The dominant contribution arises from the dimensional uncertainty of the spherical samples. The measured deviations from nominal diameter and from perfect sphericity did not exceed 1% for any of the samples listed in Table 1. Since the relative uncertainty of $\varepsilon_{r}^{\prime }$ scales as Δ$\varepsilon_{r}^{\prime }$/$\varepsilon_{r}^{\prime }$ ≈ 2 Δd/d for high-permittivity dielectric resonators, this translates to an uncertainty of approximately ±2%. The simplified electromagnetic model contributes less than 0.4% [15], and the influence of cavity dimensions is negligible because the electric energy filling factor is close to unity ($p_{e}$ for all considered configurations is in range from 0.96 up to 0.97). The combined relative uncertainty of ε_r′ is therefore estimated at ±2%.

#### 3.3.2. Dielectric Loss Tangent

The uncertainty of tan δ is governed by the Q-factor determination relative uncertainty, which increases with temperature due to resonance curve broadening and reshaping. Therefore, at room temperature this uncertainty is approximately equal to 2%, while at the highest considered temperature it is close to 10%. The contribution of conductor losses to the total uncertainty is negligible: owing to the high conductivity of the silver plating (copper cavities) and molybdenum (high-temperature cavity), combined with the high geometric factor (e.g., G ≈ 7500 Ω for the Mo cavity), the Q-factor associated with the conductive losses exceeds 10^5^ at 5 GHz [15]. Therefore, for all measurement configurations $Q_{u}^{-1}$ is at least two orders of magnitude larger than $Q_{c}^{-1}$. Consequently, even a 10% uncertainty of geometric factor determination using the simplified model reported in [15], along with the corresponding uncertainty of the $Q_{c}^{-1}$, does not propagate significantly to the tan δ uncertainty. The uncertainty associated with the filling factor determination is below 0.5% [15], which is likewise negligible in comparison to the $Q_{u}$ uncertainty. Dielectric losses of the sample supports (expanded polystyrene, single-crystal quartz, or fused silica) can be omitted in total loss analysis due to the low intrinsic loss tangent of the utilized materials and also due the to low electric energy filling factor of the support.

#### 3.3.3. Thermal Coefficient of Resonance Frequency

The general expression for the TCF of a dielectric resonator structure is given by the following expression:

(10) $$TCF=-p_{e}\alpha_{\varepsilon }/2-K_{d}\alpha \left(T\right)-K_{c}\alpha_{c}\left(T\right)$$

where $\alpha_{\varepsilon }$ is the thermal coefficient of permittivity, α(T) is the linear thermal expansion coefficient of the sample, $\alpha_{c}(T)$ is the thermal expansion coefficient of the cavity, and $K_{d}$ and $K_{c}$ are weighting factors satisfying $K_{d}\;+\;K_{c}\;=\;1$. The value of the $K_{c}$ coefficient can be calculated as:

(11) $$K_{c}=\frac{2\pi f\mu_{0}D_{sphere}}{2G}$$

where $\mu_{0}$ denotes permeability of vacuum $\mu_{0}=4\pi \;\times 10^{-7}$ H/m.

For the molybdenum cavity at room temperature ($D_{sphere}$ = 30.83 mm), $K_{c}\approx 8.5\times 10^{-2}$ and $\alpha_{c}\approx 4.8\times 10^{-6}\;K^{-1}$, yielding $K_{c}\;\alpha_{c}\approx 0.4\times 10^{-6}\;K^{-1}$ which is negligible when compared to the other terms. With $p_{e}\approx 0.97$ and $K_{d}\approx 1$, the TCF reduces to the commonly used approximation:

(12) $$TCF\approx -\frac{\alpha_{\varepsilon }}{2}-\alpha (T)$$

and the slope of f(T) directly yields the TCF as in Equation (8). It should be emphasized that TCF is a property of the entire resonant structure rather than of the material alone. The ~2.5% difference between the TCF values obtained in the copper and molybdenum cavities is consistent with the non-linearity of the thermal expansion coefficient over the broader temperature range of the Mo cavity (23–336 °C) compared to the Cu cavity (20–90 °C). The estimated uncertainty of TCF is ±3%.

#### 3.3.4. Thermal Coefficient of Permittivity

The extraction of $\alpha_{\varepsilon }$ accounts for the thermal expansion of the sample diameter via Equation (7), using the literature thermal expansion data interpolated to 3.08 mol% Y_2_O_3_ [20]. That interpolation can lead to some systematic errors, as well as, the accuracy of determination of the linear thermal expansion coefficients presented in [20] is limited. Therefore, the estimated uncertainty of $\alpha_{\varepsilon }$ is ±2–3%. Since reported $\alpha_{\varepsilon }$ values were obtained using the average slope of the real part of the permittivity in the whole considered temperature range the ~1.5% difference between the $\alpha_{\varepsilon }$ values obtained in the two cavities can be attributed to the different temperature ranges and the different sample sizes employed during the thermal experiments.

#### 3.3.5. Temperature Measurement

The temperature was monitored with an uncertainty of approximately ±1 °C (furnace thermocouple) and ±0.2 °C (environmental chamber sensor). At each temperature step, the system was allowed to stabilize for approximately 90 min prior to data acquisition, after which the resonant frequency and quality factor were measured over a 30 min time window. The results were then averaged to mitigate the impact of short-term temperature oscillations. The resulting uncertainty contribution to TCF and $\alpha_{\varepsilon }$ is negligible compared to the other sources discussed above.

## 4. Discussion

This work provides a consistent set of microwave dielectric properties of polycrystalline YSZ with 3.08 mol% Y_2_O_3_ as a function of both frequency (5–17 GHz) and temperature (20 °C to 336 °C). The key methodological contribution is the extension of dielectric resonator-based characterization, commonly applied to cylindrical or ring-shaped samples, to commercially available spherical specimens by employing an equivalent spherical enclosure model with a high electric energy filling factor in the sample ($p_{e}\approx 0.96$). High $p_{e}$ increases the sensitivity of the resonance frequency to $\varepsilon_{r}^{\prime }$ and the sensitivity of $Q_{u}$ to dielectric losses, which is the basis of accurate resonant characterization of low-loss dielectrics at microwave frequencies [13,21,22].

The validity of the simplified model is supported by two independent observations. First, the model follows from the close similarity of the electromagnetic field distributions of the TE_011_ mode in a cylindrical cavity and the TE_101_ mode in the equivalent spherical enclosure. Second, the extracted permittivity values were cross-checked using the radial mode-matching (RMM) analysis [17] (including upper- and lower-bound cylindrical approximations of the sphere), yielding $\varepsilon_{r}^{\prime }$ values within <0.7% of the simplified-model result for the tested case. This agreement, combined with the semi-analytical nature of the simplified formulation and its short computation time, makes the approach practical for routine characterization of spherical samples.

At room temperature, the extracted $\varepsilon_{r}^{\prime }$ values (Table 1) are mutually consistent, essentially frequency-independent across 5–17 GHz, and fall within the range reported for yttria-stabilized zirconia in the literature (noting that literature values vary substantially with yttria content, density/porosity, and measurement approach). They are consistent with Lanagan’s [7] work which reported that the dielectric constant of yttria-doped zirconia measured in the X-band is 34.5 (27.2) for an yttria content of 2 (9) mol%. Assuming that $\varepsilon_{r}^{\prime }$ of zirconia changes linearly in that range, for the yttria content of our samples $\varepsilon_{r}^{\prime }$ = 33.312, which is in the uncertainty range of all the results listed in Table 1. A nearly identical value of permittivity of ca. 33.3 was reported for an unspecified type of zirconia ceramic material in [23]. A permittivity value of 32.8 was reported for 3D-printed zirconia measured in the X-band [24]. The measured tan δ exhibits a slight increase with frequency, which is commonly observed in ceramic dielectrics and may reflect combined contributions of intrinsic lattice losses and defect-related mechanisms [7,21,22]. The uncertainty analysis indicates that $\varepsilon_{r}^{\prime }$ is dominated by geometric uncertainty (primarily diameter/roundness), whereas tan δ is mainly limited by the uncertainty of Q-factor determination, consistent with established resonant techniques for low-loss materials [21,22].

The temperature-dependent measurements obtained using two different cavities (Figure 5 and Figure 6) show that both $\varepsilon_{r}^{\prime }$ and tan δ increase monotonically with temperature up to 336 °C. The increase in $\varepsilon_{r}^{\prime }$ is approximately linear in both investigated temperature ranges, leading to consistent values of the thermal coefficient of permittivity $\alpha_{\varepsilon }$ (Table 2). This consistency is expected because $\alpha_{\varepsilon }$ is a material parameter, whereas the thermal coefficient of resonance frequency characterizes the entire resonant system (cavity, sample, and supports). Consequently, the observed difference in TCF between the two measurement configurations is reasonable and may reflect differences in cavity material (copper vs. molybdenum) and thermal expansion [14]. The literature on the temperature-dependent dielectric properties of YSZ is relatively sparse and virtually no comparable data has been found. AC impedance spectroscopy (10 Hz–10 MHz) of 3 mol% YSZ microcrystalline samples with ~1 µm grains was performed in [25] in the temperature range 23–450 °C. Dielectric constants were obtained from fitted impedance-arc capacitances, increasing from ca. 37 to ca. 55 between room temperature and 336 °C, which is much more than in our work which showed a small increase from ca. 33.3 to 35.5. Lanagan’s work [7] concerns measurements of YSZ up to 200 °C but they are not directly applicable as a comparison due to the 14 mol% yttria content and the fact that the measurements were made in the 100 Hz–100 kHz frequency range where the reported frequency dependence of the variations in the dielectric properties is very strong. The dielectric properties of a YSZ of 8 mol% yttria coating prepared using atmospheric plasma spraying technology were characterized in the X-band up to 900 °C [26] and $\varepsilon_{r}^{\prime }$ was found to increase from ca. 20 to 22, and tan δ from values close to 0 (below the resolution of the applied waveguide transmission-reflection method) to 0.3.

From an application perspective, the presented $\varepsilon_{r}^{\prime }(f,T)$ and tan δ(f,T) dataset reduces the spread of literature values for YSZ of similar composition and provides input parameters required for predictive design of high-temperature microwave components (e.g., dielectric resonator antennas and resonators) as well as for high-k dielectric applications where GHz-range operation and thermal loading are relevant.

Further research should address systematic characterization versus yttria content and sample density/porosity to establish composition–microstructure–property relationships. Extending the measurements to higher temperatures will require maintaining sufficient resonance Q-factor at elevated temperature and/or employing alternative resonant structures and coupling schemes. It would also be valuable to extend the dataset toward higher microwave and millimeter-wave frequencies to support emerging high-frequency applications.

## 5. Conclusions

In this work, the complex permittivity of spherical YSZ samples (3.08 mol% Y_2_O_3_) was determined at microwave frequencies in the 5–17 GHz range and over a broad temperature range from 20 °C up to over 330 °C. A dielectric resonator-based approach was extended to spherical specimens by employing an equivalent spherical enclosure model with a high electric energy filling factor ($p_{e}\approx 0.96$), enabling sensitive extraction of $\varepsilon_{r}^{\prime }$ from resonance frequency and tan δ from Q-factor data. The simplified model was validated against the radial mode-matching analysis, showing very good agreement of the extracted $\varepsilon_{r}^{\prime }$ values. At room temperature, $\varepsilon_{r}^{\prime }$ was essentially frequency-independent across 5–17 GHz, while tan δ exhibited a slight increase with frequency. Temperature-dependent measurements demonstrated a monotonic increase in both $\varepsilon_{r}^{\prime }$ and tan δ with temperature. The thermal coefficient of permittivity was consistent across two measurement configurations, yielding $\alpha_{\varepsilon }\approx 2.0\times 10^{-4}\;K^{-1}$. The resulting dataset provides a practical and reliable reference for modeling and design of high-temperature microwave components and for applications where high-permittivity dielectrics are required under thermal loading.

## Figures and Tables

**Table 1:** Results of the real part of permittivity and the dielectric loss tangent measurements at room temperature employing simplified model (equivalent enclosures of diameter $D_{sphere}$).

$f_{meas}$	$Q_{u}$	$\varepsilon_{r}^{\prime }$	$\Delta \varepsilon_{r}^{\prime }/\varepsilon_{r}^{\prime }$ (%)	tan δ	Δ(tan δ)/tan δ (%)	d (mm)	D (mm)	L (mm)	$D_{sphere}$ (mm)
5.279	1298	33.607	0.95%	$8.42\times 10^{-4}$	<2%	9.52	60	38	59.07
5.391	1250	33.573	0.95%	$8.26\times 10^{-4}$	<2%	9.52	32	21	31.83
5.423	1276	33.268	0.95%	$8.09\times 10^{-4}$	<2%	9.52	34	17	30.83
10.265 *	1054	33.510	1.27%	$9.97\times 10^{-4}$	<2%	4.95	24	12	21.76
16.711	995	33.412	1.98%	$1.04\times 10^{-3}$	<2%	3.07	12	6	10.88

* Mode-matching results: $\varepsilon_{r}^{\prime }$ = 33.7255, tan δ = $1.02\times 10^{-3}$ (average values for stacks of cylinders inscribed in the sphere and circumscribed by the sphere).

**Table 2:** Thermal coefficients of resonance frequency and permittivity obtained during thermal measurements.

Temperature Range	Cavity	TCF (1/K)	$\alpha_{\varepsilon }$ (1/K)
20–90 °C	Copper, D = 24 mm, L = 12 mm	$-9.787\times 10^{-5}$	$2.016\times 10^{-4}$
23–336 °C	Molybdenum, D = 34 mm, L = 17 mm	$-1.039\times 10^{-4}$	$2.048\times 10^{-4}$

## Data Availability

The data presented in this paper are available on request from the corresponding author.

## Abbreviations

The following abbreviations are used in this manuscript:

TCF	Thermal coefficient of resonance frequency
RMM	Radial mode-matching
